# GLI3 in cancer: a context-dependent regulator with diagnostic, prognostic, and therapeutic potential

**DOI:** 10.3389/fimmu.2026.1877245

**Published:** 2026-07-08

**Authors:** Yilin Wang, Jiayan Wu, Duocheng Zhong, Jun Xiong

**Affiliations:** 1 Department of Obstetrics and Gynecology, The Second Affiliated Hospital of Nanchang University, Nanchang, Jiangxi, China; 2 College of Ophthalmology and Optometry, Nanchang University, Nanchang, Jiangxi, China

**Keywords:** GLI3, Hedgehog signaling pathway, malignant tumors, targeted therapy, tumor biomarkers

## Abstract

**Background:**

Malignant tumors remain a major global health burden, creating an urgent need for biomarkers and therapeutic targets that can improve diagnosis, prognostic stratification, and treatment. This review focuses on glioma-associated oncogene family zinc finger 3 (GLI3), a context-dependent transcription factor in the Hedgehog signaling pathway.

**Main body:**

We integrated current evidence on GLI3 isoform processing, including the full-length activator GLI3A and the cleaved repressor GLI3R, as well as canonical and non-canonical regulatory mechanisms and the functional consequences of altered GLI3 signaling across diverse tumor types. The analysis revealed that GLI3 exerts both oncogenic and tumor-suppressive effects in a highly context-dependent manner. These effects are governed by the balance between GLI3A and GLI3R, tumor-specific signaling networks, and microenvironmental influences. Dysregulated GLI3 signaling is associated with epithelial–mesenchymal transition, cancer stem cell maintenance, immune remodeling, and therapeutic resistance. Moreover, GLI3 expression and isoform patterns show promise as diagnostic, prognostic, and susceptibility biomarkers. Emerging therapeutic strategies target GLI3 through indirect modulation, direct inhibition, or combination approaches. These findings highlight the potential clinical utility of GLI3.

**Conclusion:**

This review identifies GLI3 as a pivotal regulator of tumor biology and highlights its potential as a promising target for precision oncology. Key challenges for future research include the development of isoform-specific detection methods, clarification of non-canonical regulatory mechanisms, and improved translational validation.

## 1 Background

Malignant tumors are a major global public health challenge because of their high incidence and mortality rates. According to World Health Organization data, approximately 20 million new cancer cases and 10 million cancer-related deaths were reported worldwide in 2022. Lung cancer has the highest mortality rate among all malignancies, whereas breast cancer has the highest incidence among women (1, 2). These trends highlight the urgent need to identify novel biomarkers and develop more effective therapeutic strategies.

Glioma-associated oncogene family zinc finger 3 (GLI3) is a key transcription factor in the Hedgehog (Hh) signaling pathway and exhibits dual regulatory functions in controlling target gene expression. Upon Hh pathway activation, full-length GLI3 is converted into a transcriptional activator (GLI3A). In the absence of Hh signaling, GLI3 is processed into a transcriptional repressor (GLI3R) (3). This molecular switch may contribute to the malignant behavior of several tumor types through non-canonical Hedgehog signaling mechanisms (4, 5) and can exert oncogenic effects (6, 7). However, GLI3 may also function as a tumor suppressor in certain cancers (8). This review critically examines the molecular regulatory functions of GLI3, its distinct roles across different cancer types, and its implications for therapeutic resistance. It also evaluates the potential of GLI3 as a diagnostic biomarker and therapeutic target, thereby providing a theoretical foundation for the development of targeted interventions.

## 2 Molecular basis of GLI3

### 2.1 Structure and isoform processing

GLI3 is a key transcription factor in the Hh signaling pathway, and its functional diversity is determined by its protein structure. Similar to other GLI family members, GLI3 contains an N-terminal repressor domain, a central C2H2 zinc finger DNA-binding domain, and a C-terminal transcriptional activation domain. The zinc finger domain is particularly important because it mediates DNA recognition and transcriptional regulation (3). This structural organization enables GLI3 to function either as a transcriptional activator or as a transcriptional repressor, depending on Hh pathway activity and post-translational modifications. Both canonical SMO-dependent and non-canonical SMO-independent Hh signaling mechanisms regulate GLI3 activity (9).

**Figure 1:**
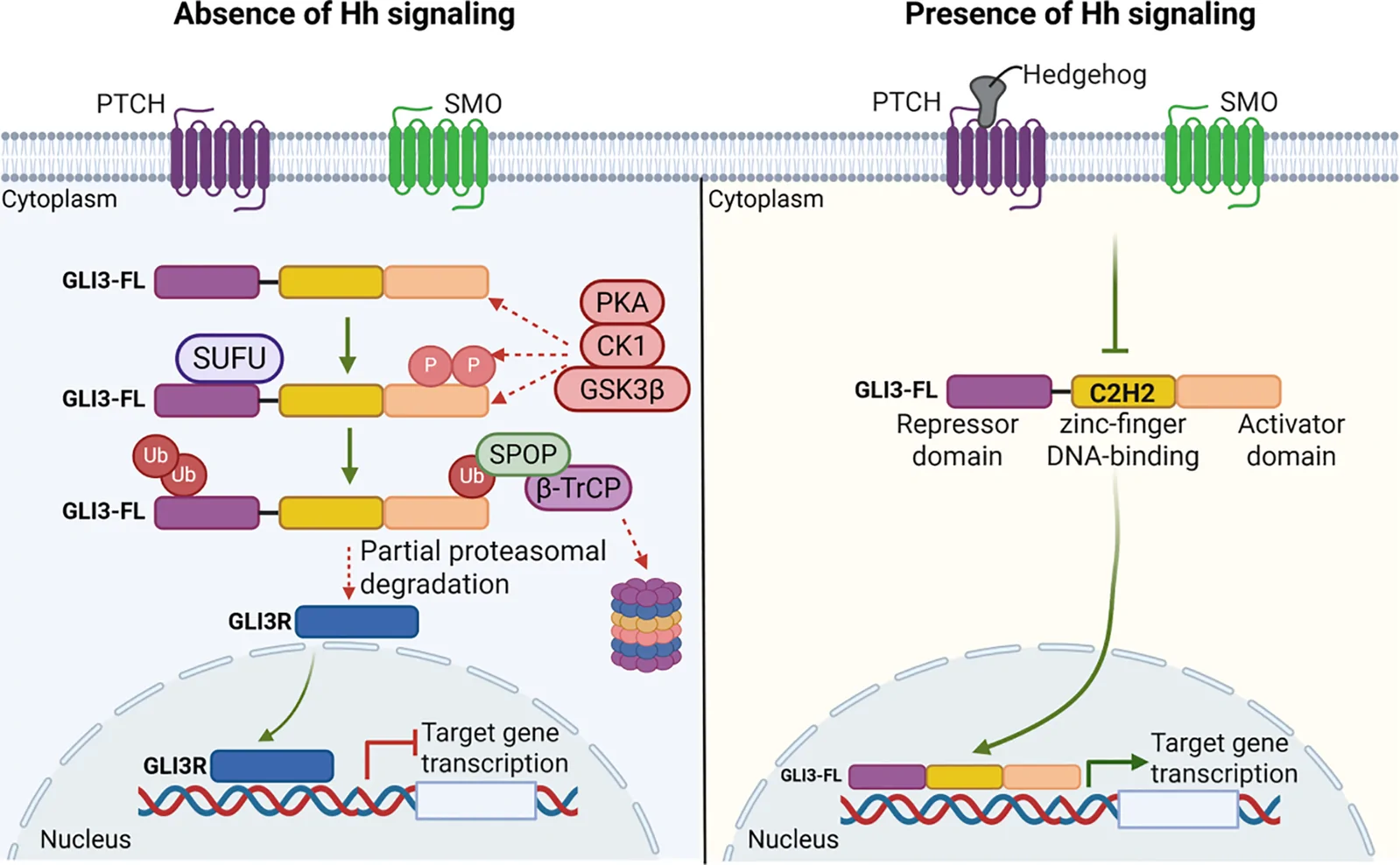
Differential regulation of GLI3 activity by Hh signaling. Left (absence of Hh): GLI3-FL is phosphorylated by PKA, CK1, and GSK3β and undergoes SPOP/β-TrCP-mediated partial proteasomal degradation to generate the repressor form, GLI3R, which inhibits transcription. Right (presence of Hh): Hh binding to PTCH relieves inhibition of SMO, thereby preventing GLI3 phosphorylation and degradation. Full-length GLI3 accumulates and functions as the nuclear activator GLI3A. This image was created by BioRender.com.

The functional heterogeneity of GLI3 is reflected in the tightly regulated generation of its isoforms through post-translational processing (**Figure 1**). In the absence of Hh signaling, full-length GLI3 (GLI3-FL) is selectively recognized by speckle-type POZ protein (SPOP), an adaptor of the cullin-3-RING E3 ubiquitin ligase complex (10, 11).Protein kinase A, casein kinase 1, and glycogen synthase kinase 3 (GSK3) subsequently phosphorylate GLI3-FL, generating a recognition signal for the SCFβ-TrCP E3 ubiquitin ligase complex. This process promotes partial proteasomal degradation of GLI3 and results in the formation of the repressor isoform, GLI3R (12, 13).In this isoform, the zinc finger DNA-binding domain is retained, whereas the C-terminal activation domain is removed. Consequently, GLI3R binds to target gene promoters and recruits co-repressors to suppress transcription. In contrast, activation of Hh signaling prevents GLI3 phosphorylation and processing, allowing GLI3-FL to accumulate and translocate to the nucleus, where it functions as the activator isoform GLI3A. Nuclear-cytoplasmic transport of GLI3 is regulated by its nuclear localization sequence and nuclear export signal (3).

The dynamic balance between GLI3A and GLI3R is an important determinant of Hh pathway output. Disruption of this balance is closely associated with abnormal cell growth and tumor progression (14).

### 2.2 Canonical and non-canonical regulation

GLI3 activity and functional output are controlled by a complex regulatory network that operates at multiple levels and involves both canonical and non-canonical signaling pathways.

In the canonical Hh pathway, the PTCH-SMO axis regulates GLI3 activity (19). In the absence of Hh signaling, suppressor of fused (SUFU) binds to GLI3, sequesters it in the cytoplasm, and facilitates its cleavage into the repressor form, GLI3R. Upon pathway activation, SMO promotes the dissociation of SUFU from GLI3 through primary cilium-dependent processes. This dissociation allows GLI3 to translocate to the nucleus and function as a transcriptional activator that induces target gene expression (16–19). Phosphorylation rapidly modulates GLI3 activity during the early stages of Hh pathway activation (15).

In addition to classical SMO-dependent regulation, SMO-independent mechanisms also contribute to the control of GLI3 activity. Oncogenic pathways such as RAS/MAPK and PI3K/AKT can directly phosphorylate and activate GLI proteins (20). Epigenetic regulation also plays an important role. For example, hypermethylation of the GLI3 promoter can result in loss of GLI3 expression and functional silencing in acute myeloid leukemia and glioblastoma (21, 22). Moreover, GSK3 exerts tumor-suppressive effects by enhancing the SUFU–GLI3 interaction and reducing GLI3 activity (23). GLI2 also positively regulates GLI3 through feedback mechanisms, thereby contributing to functional balance within the GLI signaling network (24).

Overall, GLI3 function is determined by the integrated effects of these regulatory mechanisms, which together constitute a complex molecular regulatory code. This framework may explain the substantial functional differences in GLI3 observed across tumor types (**Table 1**). Unlike GLI1, which functions exclusively as a transcriptional activator, GLI3 is capable of both transcriptional activation and repression through its unique isoform-processing mechanism. This functional versatility stems from the presence of a C-terminal repressor domain and N-terminal processing sites that are absent in GLI1. In contrast, although GLI2 possesses both activator and repressor domains, its processing into the repressor form is considerably less efficient than that of GLI3. Consequently, GLI3 stands out as the most functionally versatile member of the GLI family, with the capacity to act as either an activator or a repressor. These distinctions offer valuable insights into the isoform balance and functional redundancy that operate within the GLI family (24, 25).

**Table 1:** Interactions of GLI3 with other molecules.

Cancer type/model	Level of evidence	Interaction object	Relationship with GLI3	Molecular events/mechanisms of action	Biological significance	References
Cells (multiple tumors)	In vitro	SPOP	Upstream	Promotes GLI3 ubiquitination and partial degradation	SPOP downregulation/mutation→GLI3 stability↑	(10)
Cells (multiple tumors)	In vitro	β-TrCP (SCFβ-TrCP)	Upstream	Identify phosphorylated GLI3 and promote its degradation	Regulation of GLI3 Stability	(12)
CRPC cells	In vitro	GSK3β-GLI3-AR-V7 Axis	Upstream	GSK3β phosphorylates GLI3→GLI3R↑	Negative regulation maintains equilibrium.	(12)
Cells	In vitro	SUFU	Upstream/Collaborate	Binds to GLI3 and remains in the cytoplasm, promoting the generation of repressor proteins.	Inhibit excessive activation	(17)
Lung Adenocarcinoma/Cells & Tissue	In vitro/Animal	RAS-MAPK	Upstream	ERK pathway upregulates GLI activity/GLI3 expression	Cross-pathway activation	(20)
GC/cells	In vitro/Animal	PI3K/AKT	Upstream	Non-classical activation of GLI/ promotion of GLI3	Non-classical coupling	(20)
Liver Cancer/Fuzzy Modeling	In vitro/Animal	GLI2	Upstream	Forms a positive feedback loop with GLI3	Maintain the balance and stability of the GLI network	(24)
Cells/mouse models	In vitro/Animal	DYRK2	Upstream	DYRK2 → Phosphorylation of GLI3 → Dissociation of GLI3 from SUFU and nuclear translocation → transcriptional activity of GLI3↑	Positively regulates Hedgehog signaling	(29)
Cells	Animal	Evc/Evc2 complex	Upstream	Evc/Evc2 + Smo → Sufu/Gli3 dissociation + Gli3 transport to the ciliary tip → Regulation of GLI3 processing and activity	Evc2 mutations impair GLI3 function	(28)
Cells	In vitro/Animal	HIF-1α and ROS	Upstream	Hypoxia induces HIF-1α → Increased SHH ligand/ ROS → non-canonical Hedgehog signaling ↑→ GLI activity↑	Promotes epithelial-mesenchymal transition	(32–34)
Cells	In vitro/Animal	circular RNA, deubiquitinase, small GTPase	Upstream	Mediates the binding of OTB1 to RAB8A → RAB8A is deubiquitinated and stabilized → Primary cilia regeneration → GLI3R↑	Inhibits prostate cancer progression	(30)

*CRPC, castration-resistant prostate cancer; SPOP, speckle-type POZ protein; β-TrCP, beta-transducin repeat-containing protein; SUFU, suppressor of fused; GC, gastric cancer; HCC, hepatocellular carcinoma. Full definitions of all abbreviations are provided in Section List of abbreviations.*.

### 2.3 Regulation of GLI3 by primary cilia and microenvironmental factors

Primary cilia are essential for Hh signaling and GLI3 processing. In the absence of Hh signaling, GLI3 is phosphorylated and processed into the repressor form, GLI3R, at the ciliary base (26, 27). Upon Hh pathway activation, SMO translocates into the primary cilium and promotes GLI3 activation (28). The ciliary kinase DYRK2 directly phosphorylates GLI3, facilitating its nuclear translocation (29). In prostate cancer, regeneration of primary cilia enhances GLI3R production and suppresses tumor progression (30). Conversely, loss of primary cilia leads to uncontrolled GLI3 activation and promotes malignant progression (31).

Hypoxia modulates GLI3 activity through HIF-1α. In pancreatic cancer, hypoxia-induced HIF-1α upregulates SHH expression and promotes desmoplasia (32). In hepatocellular carcinoma, hypoxia activates non-canonical Hh signaling through reactive oxygen species (ROS), thereby promoting epithelial–mesenchymal transition (EMT) and invasion (33). In ovarian cancer stem cells, HIF-1α knockdown suppresses both the PI3K/AKT and Hh pathways (34). Metabolic stress also interacts with GLI3 signaling. In brain tumors, GLI2 and GLI3 expression correlate with the metabolic genes ENO1 and ENO2 (35). Crosstalk between Hh signaling and mTORC1 occurs within primary cilia, where AMPK and GSK3β may integrate nutrient sensing with GLI3 processing (36). These findings highlight GLI3 as a central integrator of ciliary, hypoxic, and metabolic signals within the tumor microenvironment.

## 3 Mechanisms of GLI3 in tumor development

**Figure 2:**
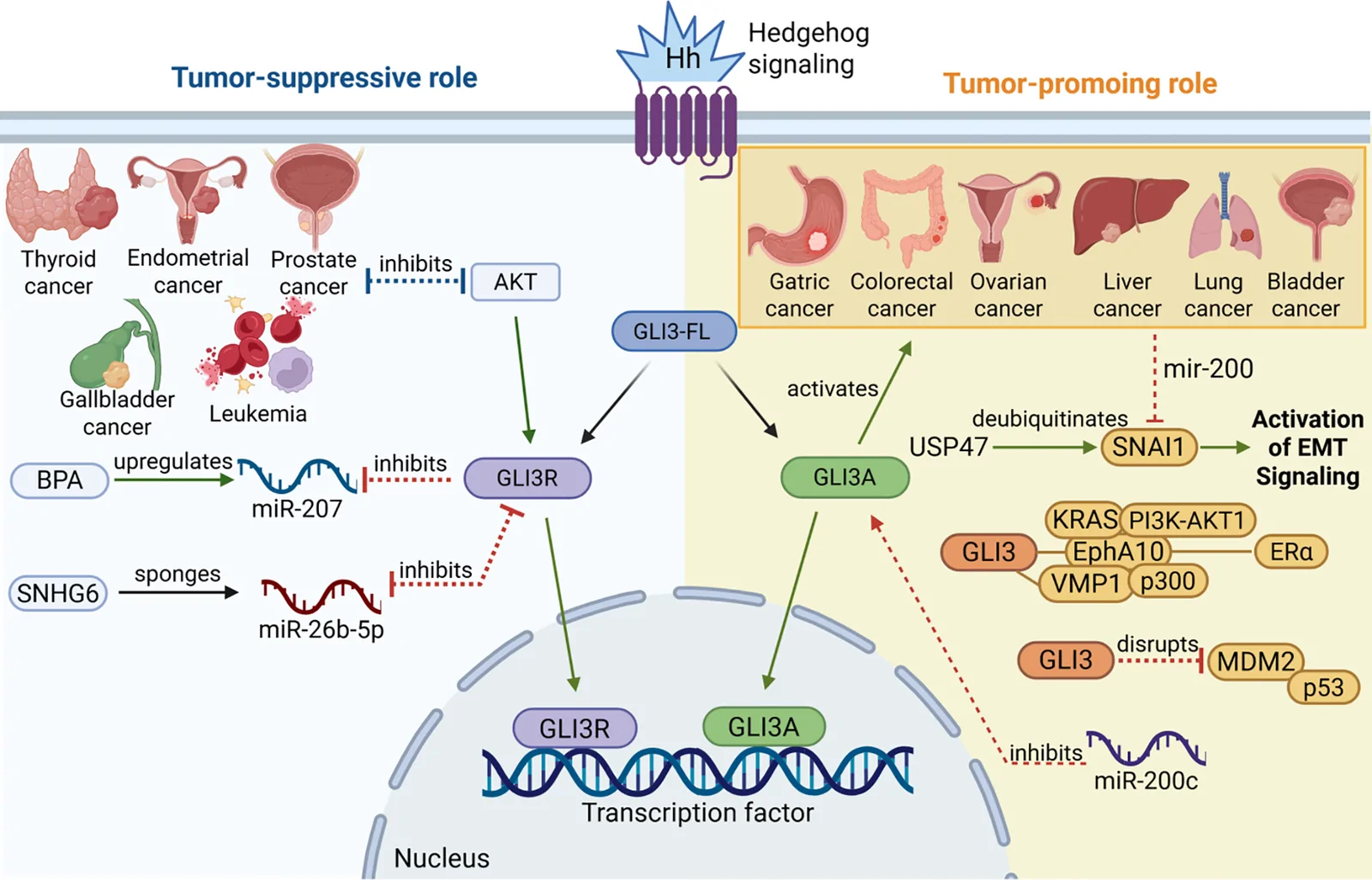
The dual regulatory role of GLI3 in tumorigenesis. The function of GLI3 depends on the tissue context and the balance between GLI3A and GLI3R. In thyroid cancer and other tumor types, GLI3R exerts tumor-suppressive effects by inhibiting AKT and other signaling pathways, and its activity is regulated by BPA, SNHG6, and other molecules. In gastric cancer and other malignancies, Hh signaling activates GLI3A and promotes tumor progression through mechanisms including epithelial–mesenchymal transition (e.g., SNAI1 activation), activation of the KRAS/PI3K pathway, and interference with p53 signaling. This image was created by BioRender.com.

GLI3 is a central transcription factor in the Hh signaling pathway and exhibits both oncogenic and tumor-suppressive functions in neoplasia. Its effects on cell proliferation and death are highly context-dependent and are influenced by the cellular environment, tissue type, the dynamic balance between GLI3A and GLI3R, and tumor-specific regulatory circuits (14). These interactions play an important role in determining tumor initiation, progression, and clinical prognosis (**Figure 2**).

### 3.1 Tumor-suppressive functions

The tumor-suppressive role of GLI3 in specific cancer types has attracted increasing attention because loss of this function is often associated with critical events in tumor progression.

Elevated GLI3 expression has been associated with a favorable prognosis in prostate cancer. In patients with hormone-naive primary prostate cancer under androgen-sufficient conditions and without prior androgen deprivation therapy, higher GLI3 expression is associated with improved clinical outcomes. Although activation of the Hh signaling pathway contributes to tumor development and metastasis, increased GLI3 expression appears to serve as an independent prognostic marker and is associated with a lower risk of disease progression (32, 37). Similarly, comparative analyses of invasive and non-invasive papillary thyroid carcinomas have shown marked downregulation of GLI3 in the invasive subtype (38). These findings suggest that physiological GLI3 expression functions as an intrinsic negative regulatory mechanism that limits the invasive and malignant potential of prostate and thyroid cancers.

In hematological malignancies, GLI3 exerts tumor-suppressive effects, particularly through SMO-independent Hh signaling. The repressor isoform GLI3R inhibits leukemia cell growth by suppressing AKT signaling, whereas epigenetic silencing of GLI3 through promoter methylation may contribute to the development of acute myeloid leukemia (22). Pharmacological studies further support the inhibitory role of GLI3. Several agents modulate GLI3-related signaling through distinct mechanisms. Niclosamide upregulates GLI3 through GSK3β inactivation, thereby suppressing Hedgehog signaling (39). Itraconazole increases GLI3 expression while reducing GLI1 and GLI2 expression, resulting in dual inhibition of the Hedgehog and Wnt pathways (40). Bozepinib upregulates the GLI3 transcriptional repressor, contributing to the elimination of cancer stem-like cells (41). In endometrial cancer, bisphenol A promotes cell proliferation by upregulating miR-107, suppressing GLI3 expression, and activating Hh signaling, further supporting the tumor-suppressive role of GLI3 (42).

The tumor-suppressive activity of GLI3 also involves complex post-transcriptional regulatory networks. In gallbladder carcinoma, a regulatory axis involving SNHG6, miR-26b-5p, and GLI3 has been identified. The oncogenic long non-coding RNA SNHG6 suppresses miR-26b-5p, which subsequently reduces GLI3 expression and attenuates its tumor-suppressive effects (39). In triple-negative breast cancer (TNBC), bioinformatic analyses indicate that GLI3 downregulation is a key molecular event. Furthermore, functional inactivation of GLI3 is associated with enhanced malignant proliferation, suggesting a tumor-suppressive role in this subtype (43).

### 3.2 Cancer-promoting functions

GLI3 promotes tumor cell growth, survival, invasion, and migration through multiple molecular pathways. Its overexpression has been associated with tumor progression, metastasis, and poor survival across several malignancies, including ovarian cancer, breast cancer, hepatocellular carcinoma, colorectal cancer, gastric cancer, and pediatric germ cell tumors (7) (44–46) (60) (47). In pediatric intracranial germ cell tumors, the transcription factor ZIC2, which modulates GLI activity, exhibits copy number gains and may function as a candidate oncogene (48). GLI3 has also been identified as a key regulator in Ewing sarcoma, where it is associated with an unfavorable prognosis and contributes to oncogenic transcriptional programs (49). In addition, environmental carcinogens such as arsenic and the intermediate filament protein nestin can disrupt the balance between GLI3A and GLI3R, resulting in aberrant Hedgehog pathway activation and promoting tumorigenesis, as observed in bladder cancer and medulloblastoma (50, 51).

In colorectal cancer, the GLI3–p300 complex participates in the KRAS-dependent PI3K/AKT1 pathway, where it regulates VMP1 expression and influences tumor cell self-renewal and survival (52). Abnormal GLI3 expression also appears to be an early molecular event in the clonal evolution of gastric adenomas. GLI3 mutations may contribute to glandular proliferation and intestinal metaplasia, suggesting a potential etiological role in gastric carcinogenesis (53–55).

GLI3 contributes to the progression of non-small cell lung cancer by promoting tumor growth and metastasis. Consistent with this observation, miR-200c directly targets and suppresses GLI3, thereby limiting its tumor-promoting activity, reducing cell viability, and inducing G0/G1 cell-cycle arrest and apoptosis (56, 57). In ERα-positive breast cancer, GLI3 enhances cell proliferation through interactions with EphA10 and regulation of ERα signaling (58, 59). Collectively, these findings suggest that the functions of GLI3 may vary across breast cancer subtypes and warrant further investigation.

Beyond DNA methylation, epitranscriptomic modifications such as N6-methyladenosine (m6A) have also been linked to GLI3 regulation. In gastric cancer, reduced m6A modification is associated with higher GLI3 mutation rates and adverse clinical outcomes, whereas m6A suppression promotes malignant phenotypes through activation of the Wnt and PI3K/AKT signaling pathways (60). In colorectal cancer, GLI3 has been reported to disrupt the MDM2–p53 interaction, thereby promoting p53 ubiquitination and degradation and weakening its tumor-suppressive function (61). However, other studies have suggested that the oncogenic effects of GLI3 may occur independently of p53 status (62).

GLI3 further promotes tumor progression by remodeling the tumor immune microenvironment and enhancing invasive behavior (**Table 2**). Mechanistically, GLI3 enhances EMT-related signaling, thereby increasing the invasive and migratory capacities of tumor cells (63). In pancreatic cancer, GLI2 and GLI3 in cancer-associated fibroblasts (CAFs) regulate chemokine and cytokine networks. They promote the expression of the immunosuppressive factors CXCL1, CXCL3, and CCL22, which recruit myeloid–derived suppressor cells (MDSCs) and tumor-associated macrophages (TAMs), while suppressing CCL5 and CXCL10, thereby limiting the recruitment of CD8^+^ T cells and natural killer (NK) cells. Knockout of GLI2 and GLI3 reduces MDSC infiltration and increases NK-cell abundance. Moreover, NK-cell ablation reverses the tumor-suppressive effects of GLI2/GLI3 deficiency, indicating that these transcription factors restrict NK-cell function through suppression of CCL5 and CXCL10. GLI2 and GLI3 also sustain IL6 and IL11 expression, promoting M2 macrophage polarization and suppressing effector T-cell activity (64). Pan-cancer analyses have revealed positive correlations between GLI3 expression and infiltration by CAFs, endothelial cells, and macrophages, whereas negative correlations have been observed with CD8^+^ T cells and NK cells (65). Through phosphorylation-sensitive ERK signaling, GLI3 expression is consistently and positively associated with EMT markers (66). In colorectal cancer, GLI3 is associated with immune-related gene signatures, including IL6ST, and poor prognosis. It also occupies a central position in the regulatory network induced by CARD11 overexpression, suggesting an important role in regulation of the tumor immune microenvironment (67, 68). In gastric cancer, GLI3 promotes tumor progression by activating USP47, which deubiquitinates SNAI1 and subsequently activates EMT signaling (5).

**Table 2:** Summary of GLI3-mediated immune regulation in cancer.

Immune cell/component	GLI3-regulated molecules/mechanisms	Functional impact	Cancer type	Reference
MDSC/TAMs	CXCL1/CXCL3/CCL22 expression ↑	Immunosuppressive myeloid cells↑	Pancreatic cancer	(64)
CD8^+^ T cells/NK cells	CCL5/CXCL10 expression↓	CD8^+^ T↓, NK cells↓ →anti-tumor immunity↓	Pancreatic cancer	(64)
Macrophages	Sustain IL6/IL11 expression	M2 polarization →effector T cells↓	Pancreatic cancer	(64)
CAFs, endothelial cells, macrophages	Positive correlation	Shape immunosuppressive microenvironment	Pan-cancer (33 types)	(65)

MDSC, myeloid-derived suppressor cell; TAM, tumor-associated macrophage; CAF, cancer-associated fibroblast; EMT, epithelial-mesenchymal transition; IL6ST, interleukin-6 signal transducer. Full definitions of all abbreviations are provided in Section List of abbreviations.*.

Further evidence indicates that GLI3 may exert pro-tumorigenic functions under androgen-deprivation conditions, such as in castration-resistant prostate cancer (CRPC) models and cell lines cultured in androgen-depleted media. Immunohistochemical studies have shown that the G protein-coupled estrogen receptor is highly expressed in prostate cancer and positively correlates with GLI3R expression and pathological grade, although the underlying regulatory mechanism remains unclear (69). Most patients with advanced prostate cancer eventually develop CRPC after endocrine therapy, which is associated with poor clinical outcomes (70). Mutation of mediator subunit 12 (MED12) enhances GLI3 activation and promotes CRPC progression (71). In addition, GLI3R has been reported to regulate both proliferation and apoptosis sensitivity in CRPC cells through the GSK3β/GLI3/AR-V7 axis (72, 73). Methodological differences further complicate the interpretation of GLI3 functions. Regarding isoform detection, studies reporting a protective role have predominantly relied on IHC or non−specific antibodies that measure total GLI3 expression, failing to distinguish GLI3-FL, t−GLI3, or GLI3R (37). In contrast, high−resolution Western blotting has revealed that accumulation of t-GLI3 or GLI3-FL, rather than total GLI3, drives tumor promotion, suggesting that technical variability may contribute to discrepant conclusions (72, 73). Regarding experimental models, tumor-suppressive effects were observed in untreated primary tumors reflecting early-stage (37), androgen-sufficient disease, whereas pro-tumorigenic roles were identified in cell lines or animal models under androgen deprivation that recapitulate late-stage CRPC (71–73). Furthermore, distinct genetic backgrounds, such as Hi-Myc, MED12 knockdown, or SPOP mutations, differentially modulate GLI3 activity (71, 72).

### 3.3 Cancer stemness and therapy tolerance

GLI3 plays an important role in maintaining stemness, self-renewal capacity, and tumorigenic potential in cancer stem cells (CSCs), thereby indirectly contributing to malignant progression (**Table 3**). The expression of GLI family members, including GLI1, GLI2, and GLI3, has been reported to correlate positively with multiple stemness-associated factors, such as SOX2, SOX9, NANOG, and POU5F1, in glioblastoma and medulloblastoma. These findings suggest that GLI3 is an important regulator of CSC maintenance in brain tumors (74). In oral squamous cell carcinoma (OSCC), Hedgehog pathway components are highly expressed in CD44-high CSCs. GLI3 depletion reduces the CSC population, inhibits tumor formation, and suppresses key stemness-associated genes, including OCT4 and BMI1, indicating that GLI3 is essential for maintaining CSC properties in OSCC (75).

**Table 3:** GLI3 and tumor stem cell stemness.

Cancer type/model	Level of evidence	Interaction object	Relationship with GLI3	Molecular events/mechanisms of action	Biological significance	References
Brain Tumors (GBM/MB, etc.)	Tissue & Cells/*In Vitro*	Stemness-Related Factors (SOX2, SOX9, NANOG, POU5F1)	Synergistic/Associated	Co-expression→Maintains CSCs	Stemness↑, Drug Resistance ↑	(70)
OSCC	*In vitro*	CSCs, stemness genes (OCT4, BMI1)	Downstream	GLI3 knockdown→CSC proportion↓, spheroid formation↓; OCT4, BMI1↓	Stemness maintenance	(75)
HCC (CD90+)	*In vitro*/Animal	IL6/JAK2/STAT3 Pathway	Upstream/Cooperative	Positive regulation of SHH/GLI axis; IL6/JAK2/STAT3-mediated	Agglomeration↑, Tumorigenicity↑	(76)
HCC	*In Vitro*	COL4A3	Upstream	COL4A3→GSK3β/Gli3/VMP1 Axis	Self-renewal↑, Migration↑, Invasion↑	(77)
PCa	*In vitro*	RRC2	Upstream	Regulates growth signaling including GLI3	Proliferation↑, Migration↑	(78)
RB	*In vitro*	miR-361-3p	Upstream	miR-361-3p→GLI1/3 →Inhibition of Hh signaling pathway	GLI3↓, Stemness↓	(79)
CRC	*In vitro*	VMP1	Downstream	Upregulation of VMP1 →Inhibition of miR-21	Malignant Phenotype Regulation (Negative Feedback)	(80)

*GBM, glioblastoma; MB, medulloblastoma; OSCC, oral squamous cell carcinoma; HCC, hepatocellular carcinoma; PCa, prostate cancer; RB, retinoblastoma; CRC, colorectal cancer; CSCs, cancer stem cells. Full definitions of all abbreviations are provided in Section List of abbreviations.

GLI3 also regulates CSC activity through interactions with other signaling pathways. In hepatocellular carcinoma, the SHH/GLI pathway positively regulates the stemness of CD90-positive liver CSCs. GLI3 expression positively correlates with CD90 levels, and GLI3 knockdown suppresses spheroid formation and tumorigenicity by inhibiting IL6/JAK2/STAT3 signaling (76). Another study showed that COL4A3 enhances the self-renewal, migration, and invasion of liver CSCs through regulation of the GSK3β/GLI3/VMP1 axis (77). In prostate cancer, RCC2 has been reported to regulate not only cell proliferation but also signaling molecules such as GLI3 (78). Non-coding RNAs may also modulate CSC stemness through GLI3-dependent mechanisms. In retinoblastoma, miR-361-3p negatively regulates Hh signaling by targeting GLI3, thereby suppressing tumor proliferation and stemness (79).

GLI3 may also indirectly influence tumor behavior through regulation of downstream target genes. For example, in colorectal cancer, GLI3 induces VMP1 expression, which negatively regulates the oncogenic microRNA miR-21 and thereby modulates tumor malignancy (80).

### 3.4 Treatment resistance

**Figure 3:**
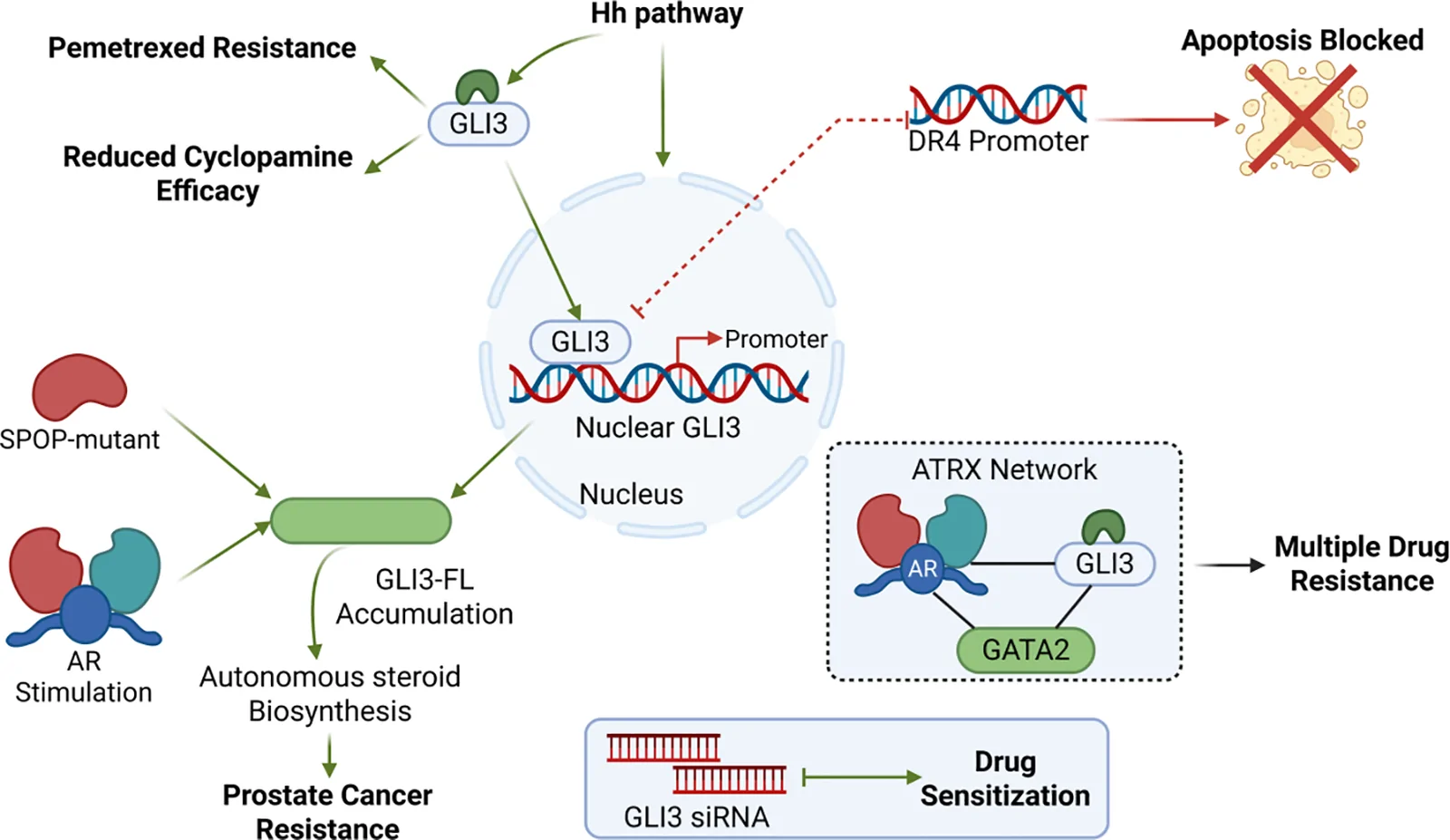
Molecular mechanisms of GLI3-mediated therapy resistance. GLI3 expression significantly influences tumor sensitivity to chemotherapeutic and targeted agents. In pancreatic and ovarian cancers, GLI3 reduces cyclopamine efficacy by suppressing apoptosis. In cholangiocarcinoma, GLI3 binds to the DR4 promoter, represses transcription, and inhibits TRAIL-induced apoptosis. Furthermore, GLI3 functions as a central node in therapy-resistance networks. It promotes multidrug resistance through the ATRX/AR/GATA2 axis in breast cancer, drives autonomous steroid biosynthesis and treatment resistance in SPOP-mutant prostate cancer, and contributes to pemetrexed resistance in non-small cell lung cancer. Conversely, GLI3-targeting siRNA can restore chemosensitivity in certain tumor types. This image was created by BioRender.com.

GLI3 expression levels substantially influence tumor cell sensitivity to targeted therapies and chemotherapeutic agents (**Figure 3**). In pancreatic and ovarian cancers, GLI3 upregulation reduces the antitumor efficacy of cyclopamine. Under these conditions, GLI3 promotes cell survival and suppresses apoptosis, thereby contributing to cyclopamine resistance (81, 82). These resistance mechanisms also involve regulation of key components of the apoptotic pathway. In cholangiocarcinoma, GLI3 directly binds to the promoter region of the death receptor DR4, represses its transcription, and inhibits tumor necrosis factor-related apoptosis-inducing ligand-induced apoptosis (83).

GLI3 also functions as an important component of broader transcriptional regulatory networks associated with therapy resistance. In HER2-negative hormone receptor-positive breast cancer, GLI3 participates in an ATRX-regulated network involving the androgen receptor, GLI3, and GATA2. Activation of this network is associated with resistance to tamoxifen, paclitaxel, and doxorubicin (84). In non-small cell lung cancer, Hedgehog signaling contributes to pemetrexed resistance, whereas modulation of GLI3 may suppress pathway activity and potentially reverse this resistance (85). In prostate cancer, SPOP mutations, together with androgen receptor activation, promote the accumulation of GLI3-FL. This accumulation drives autonomous steroid biosynthesis in tumor cells and provides a mechanistic basis for treatment resistance in SPOP-mutant prostate cancer (86).

In contrast, GLI3 inhibition can enhance chemosensitivity in certain tumor types. Studies have shown that GLI3-targeting siRNA increases the sensitivity of colon cancer cells to 5-fluorouracil and bevacizumab, thereby improving the efficacy of these treatments (61).

Notably, dysregulated autophagy is associated with a wide range of diseases, including cancer. GLI3 regulates autophagy through the AKT1–GLI3–VMP1 axis and promotes tumor cell survival, thereby providing a mechanistic explanation for GLI3-mediated therapy resistance (87). VMP1 serves as a direct downstream target of GLI3 and plays a central role in autophagy induction and the development of therapeutic resistance (52).

## 4 Clinical translation potential of GLI3

### 4.1 GLI3 as a tumor biomarker

GLI3 plays an important role in tumorigenesis, highlighting its potential both as a marker of genetic susceptibility and as a prognostic biomarker. Exome sequencing studies in individuals with a family history of lung cancer have shown that germline GLI3 mutations may significantly contribute to disease susceptibility (88). In addition, studies across multiple cancer types have linked GLI3 expression to poor clinical outcomes, suggesting its potential value as a prognostic biomarker (65).

This prognostic significance has been reported in several malignancies. In breast cancer, elevated GLI3 expression is associated with reduced recurrence-free survival in HER2-positive, progesterone receptor-negative, grade 3 tumors (22). In renal cell carcinoma, GLI3 expression is markedly increased in high-grade tumors. Moreover, high expression of the Hh ligand DHH is associated with adverse pathological features and reduced cancer-specific survival, supporting the prognostic relevance of Hh pathway activation (89).

Distinct GLI3 isoforms may also have different clinical implications. In non-small cell lung cancer, elevated expression of truncated GLI3 (t-GLI3) is significantly associated with lymph node metastasis and shorter overall survival. In lung adenocarcinoma, t-GLI3 expression has been identified as an independent prognostic factor, whereas GLI3-FL expression has also demonstrated prognostic value (90).

MicroRNA-based regulatory networks provide additional insight into the diagnostic potential of GLI3. In esophageal cancer, GLI3 has been identified as a potential target of hsa-miR-203, suggesting its involvement in carcinogenesis and its possible utility as a diagnostic biomarker (91). Additionally, in the aggressive microsatellite-stable/epithelial–mesenchymal transition subtype of gastric cancer, the EVC/EVC2/GLI3 gene expression signature has demonstrated high discriminatory accuracy, indicating potential diagnostic value (92).

### 4.2 Targeted therapeutic strategies

Because SMO-targeting Hh pathway inhibitors frequently encounter resistance, GLI3 represents an attractive downstream therapeutic target. Current GLI3-targeting approaches primarily include indirect modulation, direct inhibition, and combination strategies (**Table 4**).

**Table 4:** Therapeutic strategies targeting GLI3.

Cancer type/model	Evidence level	Target molecule/pathway	Intervention strategy	Mode of action	Primary effect/mechanism	Developmental stage	Specificity	Off-target effects	Vivo efficacy	References
CRPC	*In vitro*/Animal	GSK3β	Indirect regulation (upstream)	Inhibition	Altered GL13 processing, t-GLI3↓ suppressed tumor growth	Preclinical	non-specific	Existence (GSK3β is involved in multiple pathways)	Effective in CRPC xenograft models (combination of anti-SMO and anti-GLI1)	(72)
PC	*In Vitro*	GSK3β	Indirect Regulation (Upstream)	Inhibition	Clonidrine→p-GSK3β(Inactivated)→SUFU↑→GLI3↑→Inhibits Hh Signaling & Induces Autophagic Death	Preclinical	non-specific	Exists and is significant	Not reported	(39)
PCa	*In vitro*/Animal	SPOP-CRL3 E3 ligase	Indirect regulation (upstream)	Function restoration/enhancement	GLI3 ubiquitination↑/stability↓ inhibition of castration resistance	Preclinical	Not reported	Existence (SPOP ubiquitinates multiple substrates)	Effective in PCa xenograft models	(4)
LC	*In vitro*	α-Catenin	Indirect Regulation (Upstream)	Inhibition	Ginsenoside Rh2 phosphorylates α-catenin →Downregulates GLI3 expression→ Inhibits LC cell proliferation	Clinical	Not reported	Not reported	Not reported	(93)
Glioma	*In vitro*	GREM1	Indirect regulation (upstream)	Knockdown/inhibition	GLI3↓→EMT↓	Preclinical	Not reported	Not reported	Not reported	(94)
HNSCC	*In vitro*	GANT61 (GLI transcription complex)	Direct inhibition	Inhibition	Hh-GLI activity↓/Cell viability↓(GLI3-related)	Preclinical	Targeting the SPOP-GLI3 axis	Exists but is acceptable	Effective in various xenograft models (CRC, PC, etc.)	(97)
MM	*In vitro*/animal	GLI3R	Direct inhibition	Itraconazole treatment	GLI3R↑→Inhibition of Hh pathway	Preclinical	Not reported	Present (anti-angiogenic activity, CYP3A4 inhibition)	Effective in MM xenograft models	(40)
Cervical Cancer	*In vitro*	GLI3 (3’UTR)	Direct Inhibition (miRNA)	miR-218 mimic	GLI3↓→Proliferation/Migration↓	Preclinical	Not reported	Not reported	Not reported	(98)
BCa	*In vitro*	GLI3 signaling	Direct inhibition (natural products)	Solasonine/AlisolB23-acetate treatment	Proliferation↓, colony formation↓	Clinical	Not reported	Not reported	Not reported	(102)
CHOL	*In vitro*	GLI3	Direct inhibition	2-Methoxy-4-vinylphenol	GLI3↓→Migration↓	Preclinical	Affects GLI3 and GLI1	Not reported	Not reported	(104)
OC	*In vitro*	SMO Inhibitor (Ciclopirox) + GLI3 siRNA	Combination Therapy	Synergistic	Enhanced Apoptosis↑, Sensitization	Preclinical	Not reported	Not reported	Not reported	(82)
CHOL	*In vitro*	GLI3-targeted therapy + gemcitabine	Combination therapy	Synergistic	Synergistic antitumor effect	Preclinical	Not reported	Not reported	Not reported	(103)

*CRPC, castration-resistant prostate cancer; LC, lung cancer; PC, pancreatic cancer; PCa, prostate cancer; HNSCC, head and neck squamous cell carcinoma; MM, malignant melanoma; BCa, breast cancer; CHOL, cholangiocarcinoma; OC, ovarian cancer. Full definitions of all abbreviations are provided in Section List of abbreviations.*.

Indirect approaches target GLI3 by modulating upstream signaling pathways. In CRPC, GSK3β inhibition increases GLI3-FL levels and suppresses tumor growth (72) Similarly, niclosamide inactivates GSK3β through phosphorylation, leading to increased SUFU and GLI3 expression, negative regulation of Hh signaling, and induction of autophagic cell death (39). In prostate cancer, SPOP mutations impair GLI3 ubiquitination and degradation, thereby increasing GLI3 stability and promoting the growth of castration-resistant tumors (4). In hepatocellular carcinoma, ginsenoside Rh2 suppresses tumor proliferation by reducing GLI3 expression through α-catenin phosphorylation (93). GLI3 expression is also closely associated with EMT. Knockdown of the secreted glycoprotein GREM1 reduces GLI3 expression and thereby inhibits EMT progression (94). In breast cancer, progesterone receptor inhibitors may exert preventive effects by suppressing GLI3-mediated EMT (95). Bufalin has likewise been reported to inhibit EMT through modulation of GLI3 expression (96).

Direct-targeting approaches have enabled the development of small molecules and natural compounds that more specifically inhibit GLI3 activity. For example, GANT61 reduces cell viability in head and neck squamous cell carcinoma through inhibition of GLI3. In contrast, lithium salts increase GLI3R levels and suppress Hh pathway activity (97). Similar effects have been reported for itraconazole in malignant melanoma (40). Additionally, miR-218 (98), miR-7-5p (99), miR-506 (100), and metformin (101) have been shown to regulate GLI3 expression or activity. However, in tumor types where GLI3 functions as a tumor suppressor, these interventions may paradoxically promote tumor progression by inhibiting its protective effects. This possibility requires further investigation. In MED12-mutant breast cancer, the natural compounds solasonine and alisol B23-acetate selectively target GLI3 signaling and inhibit cancer cell proliferation (102). In cholangiocarcinoma, 2-methoxy-4-vinylphenol suppresses cancer cell migration through GLI3 downregulation (103).

Combination strategies may further enhance therapeutic efficacy through synergistic effects. In ovarian cancer, combining the SMO inhibitor cyclopamine with GLI3 gene silencing enhances anticancer activity and promotes apoptosis (82). In cholangiocarcinoma, combined treatment with gemcitabine and GLI3 targeting has also demonstrated synergistic anticancer effects (103). Effective therapeutic targeting of GLI3 may additionally require consideration of the tumor microenvironment (104). For example, co-culture with astrocytes has been shown to influence the efficacy of cyclopamine in glioblastoma models (6).

Despite these strategies, several obstacles impede clinical translation. Only SMO inhibitors have obtained FDA approval, while GLI3−directed approaches remain preclinical. No available agent selectively targets GLI3 over GLI1/GLI2; most act indirectly via upstream kinases or SMO. Off−target risks are substantial, as GSK3β and SMO regulate multiple physiological processes, and agents like niclosamide affect several pathways. *In vivo* efficacy data are limited, with most studies relying on *in vitro* models. These limitations highlight the urgent need for GLI3−selective inhibitors and rigorous *in vivo* validation.

## 5 Summary and outlook

GLI3 functions as a bidirectional transcriptional regulator in the Hh signaling pathway. By integrating both SMO-dependent and SMO-independent signals and modulating the balance between GLI3A and GLI3R, GLI3 influences key tumor-associated processes, including EMT, stemness, and therapeutic resistance. A major objective of translational cancer research is the development of precision medicine strategies. Such strategies must account for the context-dependent regulatory network of GLI3 within the tumor microenvironment. A detailed understanding of these mechanisms may facilitate the development of targeted therapies that exploit the context-dependent activity of GLI3.

To systematically assess the current state of research on GLI3 isoforms, we compiled studies cited in this review that explicitly distinguish among GLI3A, GLI3R, GLI3-FL, and t-GLI3 and summarized the detection methods used in each study (**Table 5**). This table provides an overview of the current status and limitations of isoform resolution in the field. However, several major challenges must be overcome before this mechanistic understanding can be translated into effective GLI3-targeted precision therapies.

**Table 5:** Summary of GLI3 isoform detection methods.

First author & year	Cancer type/model	GLI3 isoforms detected	Detection technique	Quantitative
Fan A et al., 2025 (30)	Prostate cancer	GLI3R	WB, IF	Yes
Kaushal JB et al., 2025 (72)	CRPC	t-GLI3	WB, MS, Co-IP	No
Zubčić V et al., 2020 (97)	HNSCC	GLI3-FL → GLI3R	WB (LiCl treatment comparison)	Yes
Li N et al., 2018 (73)	Prostate cancer	GLI3R	Co-IP, WB, IF	No
Chaudhry P et al., 2017 (22)	AML	GLI3R	Epigenetic analysis (methylation) gene expression analysis	Yes
Belgacem YH et al., 2015 (12)	Cell models	GLI3R	IHC, IF, WB	No
Bai XY et al., 2013 (90)	NSCLC	GLI3-FL & GLI3TR	IHC	Yes
Wen X et al., 2010 (27)	Cell models	GLI3-FL & t-GLI3	WB isoform-discriminating antibodies	No
Humke EW et al., 2010 (19)	Cell models	GLI3A & GLI3R	Co-IP, WB, IF	No

*GLI3-FL, full-length GLI3; GLI3R, GLI3 repressor; t-GLI3, truncated GLI3; GLI3A, GLI3 activator; WB, Western blot; IF, immunofluorescence; MS, mass spectrometry; Co-IP, co-immunoprecipitation; IHC, immunohistochemistry; CRPC, castration-resistant prostate cancer; HNSCC, head and neck squamous cell carcinoma; AML, acute myeloid leukemia; NSCLC, non-small cell lung cancer. Full definitions of all abbreviations are provided in Section List of abbreviations.

First, isoform resolution remains inadequate. Most studies measure total GLI3 expression and lack methods capable of simultaneously distinguishing among GLI3A, GLI3R, and t-GLI3. Second, the causal relationships underlying non-canonical signaling remain poorly defined. In particular, systematic evidence quantitatively characterizing GLI3 regulation through the RAS/MAPK, PI3K/AKT, and androgen receptor pathways is still lacking. Third, the contribution of the tumor microenvironment to GLI3 regulation remains unclear. Current evidence linking GLI3 regulation to cancer-associated fibroblasts, immune cells, and primary cilium status is largely correlative, and the underlying mechanisms within the tumor immune microenvironment have not been clearly established. Finally, important pharmacological challenges remain. Few small-molecule compounds directly target GLI3, and those currently available may exhibit off-target effects. In addition, potential mechanisms of acquired resistance remain largely unknown.

Future research should focus on the development of isoform-specific assays and the integration of spatial omics approaches. Furthermore, the interplay between GLI3 and TLR/NF-κB signaling, as well as its potential role in regulating PANoptosis, represents a promising direction for future investigation. The involvement of GLI3 in PANoptosis remains largely unexplored and may provide a novel conceptual framework for understanding its functions in tumor biology. Rational combination strategies should also be designed to target the GSK3β–processing–SPOP/β-TrCP–GLI3 stability axis. In addition, the EMT–stemness–immunity network should be investigated through regulatory circuits such as the USP47–SNAI1 and VMP1–miR-21 axes to further clarify the mechanistic role of GLI3 in tumor progression.

Finally, prospective studies incorporating companion biomarkers, including t-GLI3, SUFU, and primary cilia markers, together with microenvironment-based stratification approaches, are needed to evaluate the translational potential of GLI3-targeted therapies. Notably, current evidence regarding non-coding RNA-mediated regulation of GLI3 is largely limited to several microRNAs, whereas lncRNAs and circRNAs remain relatively understudied, with SNHG6 representing a notable exception. Future studies should systematically investigate these regulatory networks, as well as the epitranscriptomic modifications that modulate GLI3 function in cancer.
